# A new approach to root formation

**Published:** 2008-04-02

**Authors:** Mehdi Vatanpour, Mina Zarei, Maryam Javidi, Shiva Shirazian

**Affiliations:** 1 *Department of Endodontics, Dental School, Islamic Azad University, Tehran, Iran*; 2 *Department of Endodontics, Dental School, Mashad University of Medical Sciences. Mashad, Iran*; 3 *Specialist in Oral Medicine, Tehran, Iran*

**Keywords:** Hertwig’s root sheath, IGFs, Tooth root formation

## Abstract

In endodontics, treatment of an open apex tooth with necrotic pulp is a problem. It seems that with promotion of remnants of Hertwig’s epithelial sheath or rest of malassez accompany with a good irrigation of root canal we can expect root formation.

(Iranian Endodontic Journal 2008;3:42-43)

## Core of idea

In endodontics, we encounter with cases that they are open apex. Root canal therapy of these canals accompany with necrotic pulp is apexification apical plug (1).

The most important part of root development is Hertwig epithelial root sheath composed of inner and outer enamel epithelium (IEE and OEE). This sheath influence the adjacent mesanchymal calls to differentiate into odontoblasts.

After mineralization of first dentin matrix layer, and appearance of gaps in root sheath, mesanchymal cells from dental sac move into contact with the newly formed dentin and differentiate into cementoblasts and other calls to induce PDL (1).

Obviously the epithelial root sheath does not entirely disappear with the onset of dentinogenesis. Some cells persist within PDL and know as epithelial rests of malassez. It has been shown that some these cells retain the ability to undergo cell division such as seen in formation periradicular cyst in pathologic conditions (2).

It seems that infection and inflammation are the most important factors to stopping the proliferation activity of cells. For this reason in apexogenesis after decontamination of inflamed part of canal with a material such as Ca(OH)2 and bacteria-sealing, we observe continuing in root formation.

One of the effective manners to disinfection of the canal is copious irrigation with 5.25% NaOCl and the use of a mixture of antibiotics even without mechanical instrumentation (3).

Production of a clot in the canal to provide a matrix for the in growth of new tissue after disinfection of canal as expressed earlier, followed by a deep coronal restoration to provide a bacteria-tight seal, can probably lead to regeneration of pulp tissue such as described by Banchs and Trope (4).

Activity of cementoblasts and other regenerative cells is influenced by growth factors such as epidermal growth factor (EGF), transforming growth factor-β (TGF-β) and insulin-like growth factors (IGFs)(5).

IGFs represent a family of endocrine, paracrine and autocrine-acting polypeptide growth factors controlling pre and post natal development and growth processes.

In general, the IGF ligands, IGF-I and IGF-II are involved in various cellular process, including differentiation, proliferation, morphogenesis, growth and control of metabolic functions (6). Werner and Katz (7) have highlighted the emerging role of this growth factor system in tooth development, growth and PDL homeostasis.

IGFs are believed to behave as proliferative factors for PDL cells (8) epithelial cells of Hertwig’s root sheath (9) or cells of Malassez (10). Therefore, the IGF system plays an important role in the regulation of bone remodeling, especially in the coupling of resorption and apposition (11). Also these factors especially IGF-II and its preferential binding proteins (IGFBP-6 and IGFBP-5) have shown an involvement in the resorption repair sequence of roots (12).

Therefore it is logical that intracanal usage of solution contain high concentrate of these factors (synthetic or naturally extracted) can promote proliferation of PDL, remnant of Hertwig’s root sheath or rests of Malassez and cementoblasts. And we would be expected the interrelation signaling between external proliferated cells and in growth undifferentiated cell followed by pulp regeneration, that result in root formation.

Probably, this protocol will be on alternative treatment for immature or resorpted teeth, to prevent unwonted complication of rot shortening and difficult apical sealing.
